# Diversity analysis, nutrition, and flavor evaluation of amino acids in Chinese native geese germplasms

**DOI:** 10.14202/vetworld.2024.2932-2943

**Published:** 2024-12-26

**Authors:** Haiyue Cao, Zhenfei Yang, Ligang Wang, Xin Li, Yuanyuan Bian, Hongchang Zhao, Mengli Zhao, Xiaoming Li, Jun Wang, Guobo Sun, Shanmao Ren, Jun Yu, Huizhen Gao, Xuan Huang, Jian Wang

**Affiliations:** 1 Department of Animal Science and Technology, Jiangsu Agri-animal Husbandry Vocational College, Taizhou, 225300, China; 2 Department of Waterfowl Genetics and Breeding, National Waterfowl Gene Pool, Taizhou, 225511, China; 3 Department of Animal Science and Technology, College of Animal Science, Zijingang Campus, Zhejiang University, Hangzhou, 310058, China

**Keywords:** amino acids, diversity analysis, flavor, geese germplasms evaluation, nutrition

## Abstract

**Background and Aim:**

As living standards improve and consumption patterns shift, the market for goose meat continues to grow because of its exceptional dietary quality and distinctive flavor. The composition and content of amino acids are critical for determining the nutritional value and flavor of meat. This study aimed to evaluate the nutritional value and flavor of 10 Chinese native geese germplasms based on their amino acid content and composition.

**Materials and Methods:**

A total of 568 geese from 10 Chinese native geese germplasms reared under identical conditions were slaughtered at 10 weeks of age. The pectoralis and thigh muscles (thighs) were collected to determine the amino acid content using an amino acid analyzer. Subsequently, diversity, variance, cluster, and principal component analyses were performed to identify superior germplasm with improved nutrition and flavor.

**Results:**

The results revealed 17 amino acids in goose meat, with Glutamate and Aspartate being the most abundant. The amino acid scores of goose meat exceeded the values recommended by the Food and Agriculture Organization/World Health Organization. The Shannon–Wiener Diversity Index (1.72–2.07) indicated a high degree of diversity in amino acid content among geese germplasms. The pectoralis exhibited significantly higher amino acid content (p < 0.05 or p < 0.01) than the thigh, except for the essential amino acids to total amino acids ratio (p < 0.05 or p < 0.01). The 10 germplasms were categorized into four clusters, with Wanxi (WX) and Taizhou (TZ) geese grouped in Cluster I, displaying significantly higher nutritional value and flavor (p < 0.05 or p < 0.01) than other germplasms.

**Conclusion:**

Germplasms with superior nutritional value and flavor (WX and TZ) were identified among 10 Chinese native geese germplasms, providing valuable insights for the conservation of existing germplasms and the cultivation of new goose breeds with improved meat quality.

## Introduction

As living standards improve, consumer demand for high-quality meat products has increased, with goose meat becoming more popular because of its superior dietary quality and distinctive flavor [1]. Furthermore, as consumption patterns shift, the demand for goose meat continues to increase. Therefore, it is essential to utilize existing germplasms to develop new varieties that meet consumer demands.

The composition and content of amino acids are essential for determining the nutritional value of meat [2, 3]. As a high-quality protein source, meat shares structural and compositional similarities with human muscle and contains a balanced profile of essential amino acid (EAA) and non-EAA (NEAA) [4]. Typically, the protein quality is assessed by comparing a sample’s amino acid score (AAS) against the World Health Organization (WHO)/Food and Agriculture Organization (FAO)/United Nations University (UNU) standards [5]. The amino acids in meat are also closely associated with meat quality, particularly flavor [3]. Processes such as post-mortem aging, cooking, and curing lead to protein hydrolysis, increasing the content of free amino acids involved in Strecker and Maillard reactions, thus generating volatile flavors [6–9]. Amino acids also contribute distinct flavors, such as umami (Glutamate [Glu] and Aspartate [Asp]) and sweetness (Threonine [Thr], Serine [Ser], Glycine [Gly], and Alanine [Ala]) [10]. Various premortem factors, including breed, sex, muscle type, age, feeding system, and nutritional status, influence amino acid content [2, 11–13]. Heritable factors, especially varieties, are usually the focus of breeding programs. Consequently, screening goose germplasm with high amino acid content is crucial for cultivating new varieties with superior meat quality. Globally, China holds the largest repository of goose germplasm, which plays a vital role in biodiversity and ecosystems and serves as the foundation for sustainable waterfowl breeding [14]. Despite the abundance of native goose germplasm in China, comparative analyses of the nutritional value and flavor of many germplasms remain limited. Current studies on Chinese indigenous goose have focused primarily on the effects of various factors (e.g., feeding systems and feed additives) on specific breeds [15]. A systematic review compared the nutritional value of several Chinese native geese breeds based on data retrieval and reprocessing; however, inconsistencies in feeding methods and dietary levels across referenced studies introduced some errors in these comparisons [16]. In addition, several breeds are currently endangered due to reduced breeding numbers caused by the low economic benefits of farming and genetic dilution from the introduction of foreign species, such as Guangfeng goose (GF), Youjiang goose (YJ), and Lianhua goose (LH) [17]. Proper evaluation of germplasm resources is essential for establishing the purebred identity of endangered goose breeds, thereby contributing to their effective conservation.

Therefore, this study aimed to evaluate the nutritional value and flavor of 10 Chinese native geese germplasms raised under consistent rearing conditions based on their amino acid content and composition. The results of this study could provide valuable data for conserving existing goose germplasms and cultivating new goose breeds with better meat quality.

## Materials and Methods

### Ethical approval

This study was conducted in accordance with the Chinese Animal Welfare Guidelines and approved by the Animal Welfare Committee of Jiangsu Agri-animal Husbandry Vocational College (jsahvc-2023-22).

### Study period and location

The study was conducted from April 2022 to May 2024 at the National Waterfowl Gene Bank, Taizhou, China.

### Experimental animals and sample collection

The 10 Chinese native geese germplasms used in this study were GF, Huoyan goose (HY), LH, Sichuan goose (SC), Taihu goose (TH), Taizhou goose (TZ), Wanxi goose (WX), Xupu goose (XP), YJ, and Zhedong goose (ZD). TZ is a hybrid of TH and Rhineland geese, whereas the remaining germplasms are original Chinese varieties. Geographically, TZ, WX, ZD, TH, and HY are native to East China; GF, LH, and XP are native to Central China; and YJ and SC are native to Southwest China.

A total of 568 geese from the 10 germplasms were studied, with each group comprising half males and half females: 58 GF, 56 HY, 56 LH, 64 SC, 66 TH, 42 TZ, 52 WX, 50 XP, 56 YJ, and 68 ZD. All geese were raised under identical conditions at the National Waterfowl Gene Pool (Taizhou, China), including flat rearing, unrestricted access to water, and standardized feeding.

At 10 weeks of age, the geese were slaughtered to collect the pectoralis major (referred to as pectoralis) and gastrocnemius (referred to as thigh) for amino acid content analysis.

### Sample preparation and amino acid profile

Frozen muscle samples were thawed on ice, and 100 mg of each frozen sample was placed in a hydrolysis tube. Subsequently, 10 mL of 6 mol/L HCl was added to the tube for hydrolysis under anoxic conditions at 110°C for 23 h. After cooling, the hydrolysate was transferred to a 100-mL volumetric flask filled with ultrapure water. Next, 1 mL of the solution was vacuum-dried and re-dissolved in 1 mL of 0.2 mol/L hydrochloric acid. The solution was then filtered through a 0.22 μm filter and analyzed using an amino acid analyzer (Hitachi L-8080, Japan) for total amino acid (TAA) quantification with reference to the Amino Acids Mixture Standard Solution (Wako, Japan).

The amino acid content was calculated as follows:


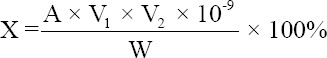


Where, X (g/100 g) is the muscle content; A (ng/μL) and V_1_ (μL) are the concentration and volume of the solution analyzed, respectively; V_2_ (mL) is the volume of the volumetric flask; and W is the weight of the hydrolyzed muscle sample. The AAS was calculated as follows [5]:


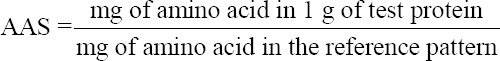


### Statistical analysis

Based on the mean observations (X̅) and standard deviation (σ), the amino acid content was classified into 10 grades according to X̅±*k*σ (*k* = 0, 0.5, 1, 1.5, 2). Then, the formula *H^'^=-ΣP_i_+inP_i_* was used to calculate the Shannon–Wiener Diversity Index based on the graded data. The *P_i_* represented the proportion of the number in grade *i* relative to the total [18].

A general linear model was applied for difference analysis using SPSS 20.0 (SPSS, Chicago, IL, USA). Based on the p-values of variance analysis (Table-S1), the linear model is as follows:


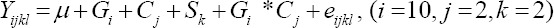


**Table-S1:** The variance analysis of three factors of amino acids (p-value).

Amino acid	Germplasm	Meat cut	Sex	Germplasmπ Meat cut
Asp	0.000	0.037	0.137	0.199
Thr	0.000	0.015	0.228	0.544
Ser	0.000	0.010	0.329	0.541
Glu	0.000	0.444	0.648	0.346
Gly	0.000	0.000	0.099	0.000
Ala	0.000	0.000	0.573	0.001
Cys	0.000	0.895	0.601	0.251
Val	0.000	0.048	0.178	0.930
Met	0.000	0.391	0.327	0.815
Ile	0.000	0.089	0.208	0.970
Leu	0.000	0.025	0.030	0.172
Tyr	0.000	0.238	0.319	0.760
Phe	0.000	0.606	0.421	0.868
Lys	0.000	0.474	0.028	0.181
His	0.000	0.981	0.664	0.320
Arg	0.000	0.000	0.111	0.010
Pro	0.000	0.000	0.377	0.001
TAA	0.000	0.002	0.279	0.110
EAA	0.000	0.129	0.076	0.722
NEAA	0.000	0.000	0.586	0.008
EAA/TAA	0.000	0.000	0.011	0.000
FAA	0.000	0.000	0.584	0.008
UTAA	0.000	0.193	0.386	0.232
STAA	0.000	0.000	0.926	0.000

Asp=Asparagine, Thr=Threonine, Ser=Serine, Glu=Glutamine, Gly=Glycine, Ala=Alanine, Cys=Cysteine, Val=Valine, Met=Methionine, Ile=Isoleucine, Leu=Leucine, Tyr=Tyrosine, Phe=Phenylalanine, Lys=Lysine, His=Histidine, Arg=Arginine, Pro=Proline, TAA=Total amino acids, EAA=Essential amino acids, EAA=Thr + Val + Met + Ile + Leu + Phe + Lys, NEAA=Non-essential amino acids, NEAA = TAA - EAA. FAA=Flavor amino acids, UTAA=Umami-taste amino acids, UTAA=Asp + Glu, STAA=Sweet-taste amino acids, STAA=Thr + Ser + Gly + Ala, FAA=UTAA + STAA. *^p^* < 0.01 indicates that the factor has a highly significant effect on amino acid content, p < 0.05 indicates a significant effect, and p > 0.05 indicates no significant effect on amino acid content

where *Y_ijkl_* = The value of amino acid content, μ = The overall mean, *G_i_* = The effect of germplasm, *C_j_* = The effect of meat cut, *S_k_* = The effect of sex, G_i_*C_j_ = The interaction of germplasm and meat cut, and *e_ijkl_* = Random residual error. Multiple comparisons among ten germplasms were adjusted using Bonferroni correction. The difference in amino acid content between meat cuts was analyzed by the independent-samples t-test.

Hierarchical cluster analysis (“hclust” function), correlation analysis (“rcorr” function), and principal component analysis (PCA, “prcomp” function) were performed using R Programming Language version 4.2.3 (R Foundation for Statistical Computing, Vienna, Austria).

After the PCA analysis, the membership function, weight calculation, and comprehensive evaluation value (*D*) were used to evaluate amino acid content comprehensively. The calculation formula is as follows [19]:


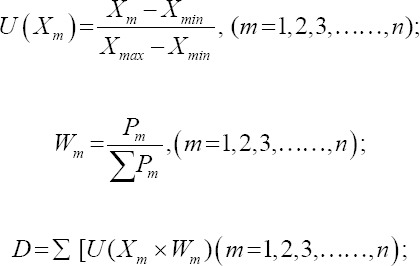


*X_m_* is the score of the m-th principal component (PC), and *X_max_* and *X_min_* are the maximum and minimum values of the m-th PC, respectively. The n is the number of extracted PCs. *W_m_* is the importance of the m-th PC in the extracted PCs; *P_m_* is the contribution rate of the m-th PC. *D* represents the comprehensive evaluation value of the amino acid content of geese germplasms.

## Results

### Diversity analysis of goose amino acid content

In this study, 17 amino acids were detected in the pectoralis and thighs of geese. As shown in Table-1, the content of Glu was the highest among the 17 amino acids, followed by Asp and Lysine (Lys), with Cysteine (Cys) being the lowest. The levels of Ser, Gly, Ala, Arginine (Arg), Proline (Pro), TAAs, NEAA, flavor amino acids (FAA), and sweet-taste amino acids (STAA) were significantly higher (p < 0.01 or p < 0.05) in the pectoralis than in the thigh. In contrast, the EAA/TAA ratio was higher (p < 0.01) in the thigh compared to the pectoralis.

**Table-1:** Diversity analysis of amino acid content in different goose meat cuts (g/100 g).

Amino acid^1^	Pectoralis							Thigh						
	
	Mean	SD	Max	Min	Range	CV (%)	*H*’^2^	Mean	SD	Max	Min	Range	CV (%)	*H*’
Asp	1.96	0.36	3.31	0.82	2.49	18.37	2.01	1.93	0.35	3.30	0.81	2.49	18.13	1.98
Thr	1.05	0.17	1.67	0.51	1.17	16.19	2.04	1.02	0.17	1.67	0.44	1.24	16.67	2.00
Ser	0.90a	0.15	1.48	0.44	1.04	16.67	2.04	0.88b	0.15	1.48	0.52	0.95	17.05	2.03
Glu	3.01	0.54	4.72	1.38	3.34	17.94	2.05	2.99	0.50	4.72	1.45	3.27	16.72	2.02
Gly	1.11^A^	0.26	2.39	0.44	1.96	23.42	1.92	0.99^B^	0.24	2.39	0.44	1.96	24.24	1.82
Ala	1.32^A^	0.23	2.30	0.54	1.76	17.42	1.98	1.26^B^	0.22	2.17	0.56	1.62	17.46	1.96
Cys	0.22	0.04	0.36	0.04	0.32	18.18	2.07	0.21	0.04	0.36	0.11	0.25	19.05	2.04
Val	1.03	0.16	1.62	0.41	1.21	15.53	2.00	1.01	0.15	1.62	0.41	1.21	14.85	1.96
Met	0.30	0.12	0.83	0.03	0.80	40.00	2.02	0.30	0.11	0.83	0.06	0.77	36.67	2.03
Ile	1.02	0.19	1.81	0.31	1.50	18.63	1.95	1.00	0.18	1.83	0.31	1.52	18.00	1.88
Leu	1.78	0.31	2.92	0.86	2.06	17.42	2.03	1.75	0.28	2.90	0.86	2.04	16.00	1.98
Tyr	0.77	0.12	1.26	0.42	0.84	15.58	2.00	0.76	0.11	1.26	0.43	0.83	14.47	2.00
Phe	1.08	0.22	2.04	0.56	1.48	20.37	1.83	1.08	0.22	2.04	0.58	1.46	20.37	1.72
Lys	1.90	0.35	3.15	0.88	2.27	18.42	2.02	1.89	0.33	3.14	0.88	2.26	17.46	1.99
His	0.61	0.11	1.13	0.30	0.83	18.03	2.03	0.60	0.11	1.13	0.31	0.81	18.33	1.95
Arg	1.50^A^	0.25	2.45	0.74	1.71	16.67	2.01	1.43^B^	0.25	2.45	0.74	1.71	17.48	1.99
Pro	0.94^A^	0.20	1.88	0.46	1.42	21.28	1.95	0.87^B^	0.20	1.89	0.46	1.43	22.99	1.90
TAA*^3^*	20.51^A^	3.03	32.57	11.86	20.71	14.77	1.99	19.97^B^	2.89	33.12	11.57	21.55	14.47	1.99
EAA^4^	8.17	1.25	12.85	3.98	8.87	15.30	2.01	8.05	1.17	13.28	4.34	8.94	14.53	2.00
NEAA^5^	12.35^A^	1.87	19.85	7.21	12.63	15.14	2.01	11.93^B^	1.79	20.08	7.03	13.04	15.00	1.99
EAA/TAA	0.398^B^	0.018	0.459	0.306	0.153	5.00	1.82	0.403^A^	0.016	0.459	0.293	0.167	5.00	1.90
FAA^6^	9.37^A^	1.48	15.13	5.47	9.66	15.80	2.03	9.07^B^	1.4	15.25	5.48	9.77	15.44	1.99
UTAA^7^	4.98	0.85	8.03	2.5	5.54	17.07	2.07	4.91	0.79	8.02	2.93	5.09	16.09	2.03
STAA^8^	4.39^A^	0.69	7.46	2.38	5.08	15.72	2.00	4.16^B^	0.67	7.23	2.39	4.84	16.11	1.96

1 Asp=Asparagine, Thr=Threonine, Ser=Serine, Glu=Glutamine, Gly=Glycine, Ala=Alanine, Cys=Cysteine, Val=Valine, Met=Methionine, Ile=Isoleucine, Leu=Leucine, Tyr=Tyrosine, Phe=Phenylalanine, Lys=Lysine, His=Histidine, Arg=Arginine, Pro=Proline,

2 *H*’=Shannon–Wiener Diversity Index,

3 TAA=Total amino acids,

4 EAA=Essential amino acids, EAA=Thr + Val + Met + Ile + Leu + Phe + Lys,

5 NEAA=Non-essential amino acids, NEAA=TAA - EAA .

6 FAA=Flavor amino acids,

7 UTAA=Umami-taste amino acids, UTAA=Asp + Glu,

8 STAA=Sweet-taste amino acids, STAA=Thr + Ser + Gly + Ala, FAA=UTAA + STAA. Means with different capital and lowercase superscript letters differ significantly at the p < 0.01 and p < 0.05 between meat cuts, respectively

With the exception of Tyrosine (Try) and the EAA/TAA ratio in pectoralis and Try, Valine (Val), TAA, EAA, and EAA/TAA ratio in the thigh, the coefficient of variation (CV) for amino acids exceeded 15%. Methionine (Met) exhibited the highest CV, followed by Gly. Amino acid diversity was evaluated using the Shannon–Wiener Diversity Index (*H*′), which ranges from 1.72 to 2.07. The diversity of amino acids in the pectoralis was generally higher than in the thigh, except Met and the EAA/TAA ratio. These findings indicate a high level of diversity in amino acid content among geese germplasms, each exhibiting distinct characteristics.

### Amino acid profile

The variance analysis revealed that the effect of meat cuts on amino acid content varied among germplasms (Table-S2). Most amino acids that exhibited significant differences between meat cuts were significantly (p < 0.05) or highly significantly (p < 0.01) higher in the pectoralis than in the thigh, including Gly in seven germplasms (GF, SC, YJ, TH, HY, LH, and XP), Ala and Pro in six germplasms (GF, SC, YJ, TH, HY, and LH), Arg in five germplasms (GF, SC, YJ, HY, LH, and XP), Lys in two germplasms (HY and LH), and Asp, Thr, Ser, Glu, Leucine (Leu), and Tyr in LH. Conversely, only the pectoralis of XP had a significantly (p < 0.05) lower Cys content than the thigh. Excluding phenylalanine (Phe) and isoleucine (Ile), WX had the highest amino acid content, followed by TZ, with significantly higher levels (p < 0.01) than those in most other germplasms. In contrast, ZD and YJ exhibited the lowest amino acid content. However, ZD exhibited the highest Phe and Ile contents, which were significantly higher (p < 0.01) than those of the other germplasms.

**Table-S2:** Amino acid content of 10 goose germplasms (MEAN ± SD, g/100 g).

Germplasm	Cut^1^	Asp	Thr	Ser	Glu	Gly	Ala	Cys	Val	Met
GF	T	1.98 ± 0.17	1.02 ± 0.09	0.88 ± 0.08	2.97 ± 0.35	0.91 ± 0.08	1.24 ± 0.11	0.23 ± 0.02	1.00 ± 0.09	0.29 ± 0.10
	P	2.06 ± 0.19	1.06 ± 0.10	0.92 ± 0.09	3.08 ± 0.39	1.10 ± 0.09**	1.34 ± 0.12**	0.22 ± 0.03	1.04 ± 0.10	0.26 ± 0.12
		2.02 ± 0.19^BC^	1.04 ± 0.09^CD^	0.90 ± 0.09^BC^	3.02 ± 0.37^CD^	1.00 ± 0.09^B^	1.29 ± 0.13^C^	0.23 ± 0.02^A^	1.02 ± 0.10^BC^	0.27 ± 0.11^DEF^
HY	T	1.89 ± 0.25	0.97 ± 0.13	0.82 ± 0.11	2.85 ± 0.42	1.03 ± 0.11	1.24 ± 0.16	0.19 ± 0.03	0.95 ± 0.12	0.33 ± 0.10
	P	1.97 ± 0.31	1.01 ± 0.17	0.87 ± 0.15	2.97 ± 0.57	1.25 ± 0.15**	1.34 ± 0.25**	0.19 ± 0.04	0.96 ± 0.19	0.31 ± 0.12
		1.93 ± 0.28^CDE^	0.99 ± 0.15^DE^	0.85 ± 0.14^CD^	2.90 ± 0.50^CDE^	1.14 ± 0.14^A^	1.29 ± 0.21^C^	0.19 ± 0.04^B^	0.95 ± 0.15^C^	0.32 ± 0.11^ABCD^
LH	T	2.03 ± 0.26	1.04 ± 0.13	0.89 ± 0.12	3.04 ± 0.49	1.06 ± 0.12	1.31 ± 0.18	0.19 ± 0.04	1.01 ± 0.12	0.33 ± 0.08
	P	2.18 ± 0.33**	1.11 ± 0.17**	0.95 ± 0.17**	3.24 ± 0.54*	1.30 ± 0.17**	1.46 ± 0.23**	0.20 ± 0.04	1.06 ± 0.17	0.33 ± 0.12
		2.10 ± 0.31^B^	1.07 ± 0.16^BC^	0.92 ± 0.15^B^	3.14 ± 0.52^BC^	1.18 ± 0.15^A^	1.38 ± 0.22^B^	0.20 ± 0.04^B^	1.04 ± 0.15^B^	0.33 ± 0.10^ABC^
SC	T	1.86 ± 0.21	0.95 ± 0.11	0.81 ± 0.11	2.85 ± 0.35	0.92 ± 0.11	1.23 ± 0.12	0.23 ± 0.03	0.98 ± 0.10	0.30 ± 0.08
	P	1.91 ± 0.26	0.98 ± 0.14	0.83 ± 0.12	2.87 ± 0.41	1.05 ± 0.12**	1.31 ± 0.16*	0.23 ± 0.04	1.01 ± 0.13	0.30 ± 0.10
		1.89 ± 0.24^DE^	0.97 ± 0.13^E^	0.82 ± 0.11^D^	2.86 ± 0.38^DE^	0.99 ± 0.11^B^	1.27 ± 0.15^C^	0.23 ± 0.03^A^	0.99 ± 0.11^BC^	0.30 ± 0.09^BCDE^
TH	T	1.87 ± 0.21	0.96 ± 0.11	0.82 ± 0.11	2.90 ± 0.35	0.92 ± 0.11	1.24 ± 0.12	0.22 ± 0.03	0.97 ± 0.09	0.30 ± 0.10
	P	1.91 ± 0.22	0.98 ± 0.12	0.83 ± 0.10	2.85 ± 0.37	1.09 ± 0.10**	1.32 ± 0.15**	0.22 ± 0.03	1.00 ± 0.11	0.31 ± 0.09
		1.89 ± 0.22^E^	0.97 ± 0.12^E^	0.82 ± 0.11^D^	2.88 ± 0.36^DE^	1.00 ± 0.11^B^	1.28 ± 0.14^C^	0.22 ± 0.03^A^	0.99 ± 0.10^BC^	0.30 ± 0.09^ABCDE^
TZ	T	2.25 ± 0.32	1.15 ± 0.16	1.01 ± 0.15	3.35 ± 0.66	1.26 ± 0.15	1.48 ± 0.22	0.23 ± 0.04	1.12 ± 0.16	0.34 ± 0.17
	P	2.23 ± 0.30	1.14 ± 0.15	1.00 ± 0.15	3.34 ± 0.69	1.21 ± 0.15	1.48 ± 0.23	0.23 ± 0.04	1.13 ± 0.18	0.35 ± 0.12
		2.24 ± 0.31^A^	1.15 ± 0.16^AB^	1.01 ± 0.15^A^	3.34 ± 0.67^AB^	1.23 ± 0.15^A^	1.48 ± 0.22^A^	0.23 ± 0.04^A^	1.13 ± 0.17^A^	0.34 ± 0.14^AB^
WX	T	2.30 ± 0.40	1.18 ± 0.20	1.02 ± 0.18	3.42 ± 0.83	1.27 ± 0.18	1.52 ± 0.26	0.23 ± 0.05	1.15 ± 0.19	0.35 ± 0.18
	P	2.27 ± 0.37	1.19 ± 0.17	1.02 ± 0.16	3.37 ± 0.84	1.21 ± 0.16	1.50 ± 0.21	0.24 ± 0.05	1.15 ± 0.15	0.37 ± 0.17
		2.28 ± 0.38^A^	1.18 ± 0.18^A^	1.02 ± 0.17^A^	3.39 ± 0.83^A^	1.24 ± 0.17^A^	1.51 ± 0.23^A^	0.23 ± 0.05^A^	1.15 ± 0.17^A^	0.36 ± 0.18^A^
XP	T	2.04 ± 0.15	1.05 ± 0.08	0.91 ± 0.07	3.04 ± 0.32	0.95 ± 0.07	1.29 ± 0.10	0.23 ± 0.02*	1.04 ± 0.08	0.30 ± 0.11
	P	2.04 ± 0.16	1.06 ± 0.08	0.91 ± 0.08	3.01 ± 0.33	1.09 ± 0.08**	1.34 ± 0.10	0.22 ± 0.03	1.04 ± 0.08	0.27 ± 0.12
		2.04 ± 0.15^BC^	1.06 ± 0.08^CD^	0.91 ± 0.07^BC^	3.02 ± 0.33^CD^	1.02 ± 0.07^B^	1.31 ± 0.10^BC^	0.22 ± 0.02^A^	1.04 ± 0.08^B^	0.28 ± 0.11^CDE^
YJ	T	1.83 ± 0.22	0.93 ± 0.11	0.79 ± 0.11	2.80 ± 0.44	0.89 ± 0.11	1.20 ± 0.11	0.22 ± 0.03	0.94 ± 0.09	0.27 ± 0.10
	P	1.88 ± 0.22	0.96 ± 0.11	0.81 ± 0.10	2.83 ± 0.40	1.12 ± 0.10**	1.28 ± 0.13*	0.23 ± 0.04	0.95 ± 0.10	0.25 ± 0.10
		1.86 ± 0.22^E^	0.95 ± 0.11^E^	0.80 ± 0.11^D^	2.82 ± 0.42^DE^	1.01 ± 0.11^B^	1.24 ± 0.13^C^	0.22 ± 0.03^A^	0.95 ± 0.10^C^	0.26 ± 0.10^EF^
ZD	T	1.42 ± 0.30	1.04 ± 0.25	0.93 ± 0.20	2.84 ± 0.32	0.86 ± 0.20	0.99 ± 0.21	0.19 ± 0.02	1.03 ± 0.25	0.22 ± 0.05
	P	1.38 ± 0.26	1.03 ± 0.23	0.93 ± 0.19	2.74 ± 0.32	0.81 ± 0.19	0.96 ± 0.17	0.19 ± 0.02	1.02 ± 0.24	0.23 ± 0.05
		1.40 ± 0.28^F^	1.03 ± 0.24^CD^	0.93 ± 0.20^B^	2.79 ± 0.32^E^	0.83 ± 0.20^C^	0.97 ± 0.19^D^	0.19 ± 0.02^B^	1.03 ± 0.24^B^	0.23 ± 0.05^F^

**Germplasm**	**Cut^1^**		**Ile**	**Leu**	**Tyr**	**Phe**	**Lys**	**His**	**Arg**	**Pro**

GF	T		0.98 ± 0.09	1.79 ± 0.16	0.78 ± 0.07	1.01 ± 0.10	1.95 ± 0.17	0.61 ± 0.08	1.42 ± 0.12	0.80 ± 0.07
	P		1.01 ± 0.10	1.86 ± 0.18	0.79 ± 0.08	1.04 ± 0.10	1.99 ± 0.19	0.60 ± 0.09	1.52 ± 0.13*	0.89 ± 0.09**
			0.99 ± 0.09^CD^	1.82 ± 0.17^B^	0.79 ± 0.07^C^	1.03 ± 0.10^DE^	1.97 ± 0.19^BCD^	0.60 ± 0.09^CDE^	1.47 ± 0.14^BC^	0.84 ± 0.10^B^
HY	T		0.91 ± 0.12	1.67 ± 0.22	0.73 ± 0.13	1.03 ± 0.29	1.74 ± 0.25	0.59 ± 0.11	1.29 ± 0.24	0.89 ± 0.20
	P		0.93 ± 0.19	1.73 ± 0.32	0.73 ± 0.14	0.99 ± 0.22	1.85 ± 0.31*	0.56 ± 0.15	1.44 ± 0.27*	1.01 ± 0.26**
			0.92 ± 0.16^D^	1.70 ± 0.27^C^	0.73 ± 0.13^D^	1.01 ± 0.26^DE^	1.79 ± 0.28^E^	0.58 ± 0.14^DE^	1.36 ± 0.26^CD^	0.95 ± 0.24^A^
LH	T		0.97 ± 0.12	1.80 ± 0.23	0.77 ± 0.10	1.04 ± 0.17	1.94 ± 0.26	0.60 ± 0.12	1.43 ± 0.21	0.93 ± 0.17
	P		1.03 ± 0.17	1.92 ± 0.29**	0.81 ± 0.12*	1.08 ± 0.16	2.05 ± 0.33*	0.62 ± 0.17	1.59 ± 0.26*	1.07 ± 0.24**
			1.00 ± 0.15^CD^	1.86 ± 0.27^B^	0.79 ± 0.11^C^	1.06 ± 0.17^BCD^	2.00 ± 0.31^B^	0.61 ± 0.15^CDE^	1.51 ± 0.25^B^	1.00 ± 0.21^A^
SC	T		0.95 ± 0.10	1.73 ± 0.18	0.72 ± 0.08	1.00 ± 0.09	1.85 ± 0.21	0.56 ± 0.11	1.31 ± 0.17	0.75 ± 0.10
	P		0.98 ± 0.13	1.79 ± 0.23	0.74 ± 0.10	1.03 ± 0.13	1.87 ± 0.26	0.58 ± 0.12	1.40 ± 0.19*	0.82 ± 0.11*
			0.97 ± 0.11^CD^	1.76 ± 0.21^BC^	0.73 ± 0.09^D^	1.02 ± 0.11^DE^	1.86 ± 0.24^CDE^	0.57 ± 0.11^EF^	1.36 ± 0.19^D^	0.79 ± 0.11^B^
TH	T		0.95 ± 0.09	1.73 ± 0.17	0.73 ± 0.08	1.00 ± 0.09	1.86 ± 0.21	0.56 ± 0.11	1.32 ± 0.17	0.76 ± 0.10

**Germplasm**	**Cut^1^**		**Ile**	**Leu**	**Tyr**	**Phe**	**Lys**	**His**	**Arg**	**Pro**

	P		0.97 ± 0.10	1.77 ± 0.19	0.73 ± 0.09	1.02 ± 0.10	1.85 ± 0.22	0.57 ± 0.10	1.40 ± 0.17	0.84 ± 0.16**
			0.96 ± 0.10^CD^	1.75 ± 0.18^BC^	0.73 ± 0.08^D^	1.01 ± 0.10^DE^	1.85 ± 0.22^DE^	0.56 ± 0.11^EF^	1.36 ± 0.17^D^	0.80 ± 0.14^B^
TZ	T		1.09 ± 0.17	1.98 ± 0.29	0.85 ± 0.12	1.13 ± 0.15	2.17 ± 0.32	0.67 ± 0.15	1.65 ± 0.24	1.03 ± 0.20
	P		1.11 ± 0.19	1.99 ± 0.30	0.85 ± 0.13	1.14 ± 0.17	2.18 ± 0.30	0.67 ± 0.15	1.64 ± 0.24	1.00 ± 0.16
			1.10 ± 0.18^AB^	1.99 ± 0.30^A^	0.85 ± 0.13^AB^	1.14 ± 0.16^BC^	2.17 ± 0.31^A^	0.67 ± 0.15^AB^	1.65 ± 0.24^A^	1.02 ± 0.18^A^
WX	T		1.12 ± 0.20	2.03 ± 0.35	0.87 ± 0.15	1.15 ± 0.19	2.21 ± 0.40	0.68 ± 0.18	1.67 ± 0.30	1.04 ± 0.22
	P		1.11 ± 0.15	2.02 ± 0.29	0.88 ± 0.13	1.15 ± 0.16	2.18 ± 0.37	0.69 ± 0.16	1.65 ± 0.26	1.03 ± 0.18
			1.11 ± 0.18^A^	2.02 ± 0.32^A^	0.88 ± 0.14^A^	1.15 ± 0.18^B^	2.19 ± 0.38^A^	0.69 ± 0.17^A^	1.66 ± 0.28^A^	1.04 ± 0.20^A^
XP	T		1.02 ± 0.08	1.85 ± 0.14	0.81 ± 0.06	1.05 ± 0.09	2.01 ± 0.15	0.63 ± 0.07	1.47 ± 0.11	0.83 ± 0.07
	P		1.01 ± 0.08	1.86 ± 0.15	0.79 ± 0.06	1.04 ± 0.08	1.97 ± 0.16	0.61 ± 0.08	1.51 ± 0.10	0.89 ± 0.07
			1.01 ± 0.08^BC^	1.85 ± 0.14^B^	0.80 ± 0.06^BC^	1.05 ± 0.08^CDE^	1.99 ± 0.15^BC^	0.62 ± 0.07^BCD^	1.49 ± 0.11^B^	0.86 ± 0.07^B^
YJ	T		0.93 ± 0.09	1.68 ± 0.18	0.73 ± 0.08	0.96 ± 0.09	1.84 ± 0.22	0.53 ± 0.11	1.30 ± 0.15	0.72 ± 0.09
	P		0.93 ± 0.10	1.70 ± 0.19	0.72 ± 0.08	0.94 ± 0.09	1.80 ± 0.22	0.52 ± 0.10	1.40 ± 0.16*	0.86 ± 0.13**
			0.93 ± 0.10^CD^	1.69 ± 0.19^C^	0.72 ± 0.08^D^	0.95 ± 0.09^E^	1.82 ± 0.22^E^	0.53 ± 0.11^F^	1.35 ± 0.16^D^	0.79 ± 0.13^B^
ZD	T		1.14 ± 0.35	1.38 ± 0.27	0.71 ± 0.09	1.35 ± 0.37	1.49 ± 0.30	0.64 ± 0.20	1.52 ± 0.35	0.98 ± 0.29
	P		1.15 ± 0.36	1.32 ± 0.22	0.71 ± 0.11	1.36 ± 0.41	1.43 ± 0.26	0.63 ± 0.19	1.50 ± 0.39	0.98 ± 0.30
			1.14 ± 0.35^A^	1.35 ± 0.25^D^	0.71 ± 0.10^D^	1.36 ± 0.39^A^	1.46 ± 0.28^F^	0.64 ± 0.20^BC^	1.51 ± 0.37^B^	0.98 ± 0.29^A^

1 T, Thigh; P, Pectoralis. Asp=Asparagine, Thr=Threonine, Ser=Serine, Glu=Glutamine, Gly=Glycine, Ala=Alanine, Cys=Cysteine, Val=Valine, Met=Methionine, Ile=Isoleucine, Leu=Leucine, Tyr=Tyrosine, Phe=Phenylalanine, Lys=Lysine, His=Histidine, Arg=Arginine, Pro=Proline. GF=Guangfeng goose, HY=Huoyan goose, LH=Lianhua goose, SC=Sichuan goose, TH=Taihu goose, TZ=Taizhou goose, WX=Wanxi goose, XP=Xupu goose, YJ=Youjiang goose, ZD=Zhedong goose.

* and

** Indicate that there are significant differences between different meat cuts within the same germplasm at p < 0.05 and p < 0.01, respectively. Means with different capital superscripts differ significantly at the p < 0.01 among different germplasms; means with the same letter do not differ significantly (p > 0.05).

### Amino acid nutrition and flavor evaluation

As shown in Table-2, the content of TAA in two germplasms (HY and LH), NEAA in four germplasms (GF, YJ, HY, and LH), FAA in three germplasms (GF, HY, and LH), STAA in six germplasms (GF, SC, YJ, TH, HY, and LH), and EAA, umami-taste amino acids (UTAA) in LH were significantly (p < 0.05) or highly significantly (p < 0.01) higher in the pectoralis than in the thigh. Conversely, in GF, YJ, HY, and LH, the EAA/TAA ratio wassignificantly (p < 0.05) or highly significantly (p < 0.01) higher in the thigh compared to the pectoralis. Regarding differences among germplasms, the contents of TAA, EAA, NEAA, and FAA in WX and TZ were significantly higher (p < 0.01) than those in other germplasms, whereas the EAA/TAA ratio in ZD and SC was significantly higher (p < 0.01) than that in the other germplasms.

**Table-2:** EAA, NEAA, and FAA content of 10 goose germplasms (mean ± SD, g/100 g).

Germplasm	Cut^1^	TAA^2^	EAA^3^	NEAA*^4^*	EAA/TAA	FAA^5^	UTAA^6^	STAA^7^
GF	T	19.85 ± 1.78	8.04 ± 0.71	11.82 ± 1.09	0.4049 ± 0.0067*	9.00 ± 0.86	4.95 ± 0.51	4.05 ± 0.36
	P	20.77 ± 1.96	8.26 ± 0.80	12.51 ± 1.19*	0.3975 ± 0.0085	9.55 ± 0.94*	5.13 ± 0.57	4.42 ± 0.40**
		20.31 ± 1.92^BC^	8.15 ± 0.76^BCD^	12.17 ± 1.19^BCD^	0.4011 ± 0.0085^ABC^	9.28 ± 0.93^BC^	5.04 ± 0.55^CD^	4.23 ± 0.42^CD^
HY	T	19.12 ± 2.46	7.59 ± 1.02	11.53 ± 1.53	0.3971 ± 0.0170**	8.80 ± 1.15	4.74 ± 0.65	4.06 ± 0.56
	P	20.09 ± 3.56*	7.77 ± 1.44	12.32 ± 2.20**	0.3868 ± 0.0185	9.39 ± 1.64**	4.93 ± 0.86	4.46 ± 0.84**
		19.59 ± 3.07^CD^	7.68 ± 1.24^DE^	11.91 ± 1.91^CDE^	0.3921 ± 0.0184^D^	9.09 ± 1.43^CD^	4.83 ± 0.76^DE^	4.26 ± 0.73^CD^
LH	T	20.38 ± 2.60	8.14 ± 1.03	12.24 ± 1.61	0.3995 ± 0.0104**	9.37 ± 1.26	5.07 ± 0.73	4.30 ± 0.57
	P	22.00 ± 3.35**	8.58 ± 1.35*	13.42 ± 2.06**	0.3899 ± 0.0150	10.24 ± 1.58**	5.42 ± 0.84**	4.82 ± 0.80**
		21.19 ± 3.10^B^	8.36 ± 1.22^B^	12.83 ± 1.93^B^	0.3947 ± 0.0137^CD^	9.80 ± 1.49^B^	5.24 ± 0.80^BC^	4.56 ± 0.74^B^
SC	T	19.01 ± 2.05	7.76 ± 0.82	11.25 ± 1.26	0.4085 ± 0.0088	8.62 ± 0.95	4.71 ± 0.55	3.91 ± 0.43
	P	19.71 ± 2.56	7.96 ± 1.06	11.75 ± 1.51	0.4036 ± 0.0088	8.97 ± 1.15	4.79 ± 0.67	4.18 ± 0.51**
		19.36 ± 2.34^CD^	7.86 ± 0.95^BCDE^	11.50 ± 1.41^DEF^	0.4060 ± 0.0091^A^	8.79 ± 1.07^CD^	4.75 ± 0.61^DE^	4.05 ± 0.49^CD^
TH	T	19.11 ± 2.01	7.78 ± 0.78	11.34 ± 1.26	0.4070 ± 0.0094	8.71 ± 0.96	4.77 ± 0.55	3.94 ± 0.43
	P	19.66 ± 2.16	7.90 ± 0.88	11.76 ± 1.35	0.4022 ± 0.0133	8.97 ± 1.02	4.76 ± 0.57	4.21 ± 0.50**
		19.39 ± 2.10^CD^	7.84 ± 0.83^CDE^	11.55 ± 1.32^CDEF^	0.4046 ± 0.0117^AB^	8.84 ± 0.99^CD^	4.76 ± 0.56^DE^	4.07 ± 0.48^CD^
TZ	T	22.78 ± 3.36	8.99 ± 1.33	13.79 ± 2.08	0.3947 ± 0.0126	10.51 ± 1.62	5.60 ± 0.94	4.91 ± 0.75
	P	22.71 ± 3.29	9.04 ± 1.33	13.66 ± 2.01	0.3984 ± 0.0116	10.40 ± 1.55	5.57 ± 0.94	4.83 ± 0.68
		22.74 ± 3.31^A^	9.02 ± 1.33^A^	13.72 ± 2.03^A^	0.3967 ± 0.0122^BCD^	10.45 ± 1.58^A^	5.59 ± 0.94^AB^	4.87 ± 0.71^A^
WX	T	23.20 ± 4.05	9.18 ± 1.62	14.02 ± 2.50	0.3959 ± 0.0171	10.70 ± 1.95	5.72 ± 1.18	4.98 ± 0.85
	P	23.02 ± 3.39	9.15 ± 1.28	13.87 ± 2.17	0.3981 ± 0.0150	10.56 ± 1.80	5.64 ± 1.17	4.92 ± 0.71
		23.11 ± 3.72^A^	9.16 ± 1.45^A^	13.94 ± 2.33^A^	0.3970 ± 0.0160^BCD^	10.63 ± 1.87^A^	5.68 ± 1.17^A^	4.95 ± 0.78^A^
XP	T	20.52 ± 1.56	8.32 ± 0.66	12.20 ± 0.92	0.4053 ± 0.0074	9.28 ± 0.72	5.08 ± 0.45	4.19 ± 0.31
	P	20.63 ± 1.55	8.25 ± 0.64	12.38 ± 0.94	0.3997 ± 0.0079	9.43 ± 0.75	5.04 ± 0.47	4.39 ± 0.31
		20.58 ± 1.55^BC^	8.28 ± 0.65^BC^	12.30 ± 0.93^BC^	0.4025 ± 0.0081^ABC^	9.35 ± 0.74^BC^	5.06 ± 0.46^CD^	4.29 ± 0.32^BC^
YJ	T	18.56 ± 2.06	7.55 ± 0.78	11.01 ± 1.30	0.4071 ± 0.0088**	8.45 ± 1.01	4.64 ± 0.65	3.81 ± 0.38
	P	19.18 ± 2.15	7.54 ± 0.88	11.64 ± 1.33*	0.3931 ± 0.0130	8.87 ± 1.02	4.71 ± 0.60	4.17 ± 0.47**
		18.87 ± 2.12^D^	7.54 ± 0.83^E^	11.33 ± 1.35^EF^	0.4001 ± 0.0131^ABC^	8.66 ± 1.03^D^	4.67 ± 0.62^E^	3.99 ± 0.46^DE^
ZD	T	18.72 ± 2.45	7.66 ± 1.45	11.06 ± 1.20	0.4067 ± 0.0325	8.07 ± 0.90	4.26 ± 0.37	3.81 ± 0.69
	P	18.38 ± 2.29	7.55 ± 1.40	10.83 ± 1.08	0.4077 ± 0.0354	7.85 ± 0.75	4.12 ± 0.40	3.73 ± 0.55
		18.55 ± 2.37^D^	7.60 ± 1.42^E^	10.95 ± 1.14^F^	0.4072 ± 0.0339^A^	7.96 ± 0.83^F^	4.19 ± 0.39^F^	3.77 ± 0.62^E^

1 T=Thigh, P=Pectoralis,

2 TAA=Total amino acids,

3 EAA=Essential amino acids, EAA=Thr + Val + Met + Ile + Leu + Phe + Lys.

4 NEAA=Non-essential amino acids, NEAA=TAA – EAA,

5 FAA=Flavor amino acids,

6 UTAA=Umami-taste amino acids, UTAA=Asp + Glu,

7 STAA=Sweet-taste amino acids, STAA=Thr + Ser + Gly + Ala, FAA=UTAA + STAA, GF=Guangfeng goose, HY=Huoyan goose, LH=Lianhua goose, SC=Sichuan goose, TH=Taihu goose, TZ=Taizhou goose, WX=Wanxi goose, XP=Xupu goose, YJ=Youjiang goose, ZD=Zhedong goose.

* and

** indicate significant (p < 0.05) and highly significant differences (p < 0.01) between different meat cuts of the same germplasm, respectively. Means with different capital superscripts differ significantly at the p < 0.01 in different germplasms, means with the same letter do not differ significantly (p > 0.05)

The AAS was calculated (Table-3) to assess the nutritional value of goose protein. All AAS values for the 10 germplasms exceeded 100, indicating that goose meat has a high nutritional value. Both the pectoralis and thigh scored the highest for Phe + Tyr and the lowest for Met + Cys. In TZ, WX, and ZD, the AAS of the pectoralis surpassed that of the thigh (with exceptions for Thr in TZ and Leu and Cys in WX and ZD), whereas in other germplasms (except for Lys in HY and Histidine in SC), it was lower. SC, GF, and TH achieved the highest scores for Leu, Lys, and Met, whereas ZD recorded the highest scores for the remaining amino acids.

**Table-3:** EAA score of the goose.

Germplasm	Cut^1^	His	Ile	Leu	Lys	Met + Cys	Phe + Tyr	Thr	Val
GF	T	203	164	152	218	118	238	224	129
	P	194	162	152	213	106	232	222	128
		199	163	152	215	112	235	223	129
HY	T	207	158	148	203	123	243	219	127
	P	185	154	146	204	114	225	218	123
		196	156	147	203	118	234	219	125
LH	T	196	159	149	212	117	233	221	128
	P	189	156	148	207	110	226	220	123
		192	157	149	209	114	229	221	125
SC	T	196	167	154	216	125	239	217	132
	P	196	166	154	211	121	236	217	131
		196	167	154	214	123	238	217	131
TH	T	194	166	154	216	125	238	217	131
	P	194	165	153	209	122	235	216	131
		194	166	153	213	123	236	217	131
TZ	T	195	159	148	211	114	229	220	126
	P	198	162	149	213	115	232	218	128
		196	161	148	212	114	231	219	127
WX	T	196	160	149	211	113	229	221	127
	P	201	161	148	210	119	232	224	128
		198	161	148	211	116	231	223	127
XP	T	206	165	153	217	117	239	223	130
	P	197	163	152	213	107	234	223	129
		201	164	153	215	112	237	223	130
YJ	T	190	166	153	220	119	240	219	131
	P	181	162	150	208	113	228	217	127
		186	164	152	214	116	234	218	129
ZD	T	229	203	125	177	100	290	241	141
	P	230	208	122	173	102	297	244	143
		229	206	124	175	101	293	242	142

1 T=Thigh, P=Pectoralis, His=Histidine, Ile=Isoleucine, Leu=Leucine, Lys=Lysine, Met=Methionine, Cys=Cysteine, Phe=Phenylalanine, Tyr=Tyrosine, Thr=Threonine, Val=Valine, GF=Guangfeng goose, HY=Huoyan goose, LH=Lianhua goose, SC=Sichuan goose, TH=Taihu goose, TZ=Taizhou goose, WX=Wanxi goose, XP=Xupu goose, YJ=Youjiang goose, ZD=Zhedong goose

### Cluster analysis

**Figure-1:**
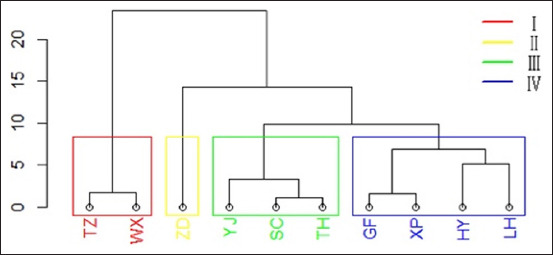
Cluster dendrogram of 10 goose germplasms. GF=Guangfeng goose, HY=Huoyan goose, LH=Lianhua goose, SC=Sichuan goose, TH=Taihu goose, TZ=Taizhou goose, WX=Wanxi goose, XP=Xupu goose, YJ=Youjiang goose, ZD=Zhedong goose.

This study employed hierarchical clustering to examine the relationships among 10 geese germplasms according to amino acid content. The results revealed that these germplasms can be categorized into four main groups, as detailed in Figure-1 and Table-S3. The results indicate that most geographically close germplasms are grouped together. Cluster I (East China) comprised WX and TZ, which exhibited the highest amino acid content, excluding Ile and Phe. ZD, with elevated levels of Ile and Phe, formed an independent Cluster II (East China). TH (East China) was grouped into Cluster III with YJ and SC (Southwest China). Finally, HY (East China) was clustered to the cluster IV with GF, XP, and LH (Central China).

**Table-S3:** Amino acid content of four groups of goose germplasms (MEAN: g/100 g).

Amino acid	I	II	III	IV	Amino acid	I	II	III	IV
P_Asp	2.26	1.38	1.90	2.06	T_Asp	2.28	1.42	1.86	1.99
P_Thr	1.17	1.03	0.97	1.06	T_Thr	1.17	1.04	0.95	1.02
P_Ser	1.02	0.93	0.82	0.91	T_Ser	1.01	0.93	0.80	0.88
P_Glu	3.37	2.74	2.85	3.08	T_Glu	3.39	2.84	2.85	2.98
P_Gly	1.20	0.81	1.09	1.19	T_Gly	1.27	0.86	0.91	0.99
P_Ala	1.49	0.96	1.31	1.37	T_Ala	1.50	0.99	1.23	1.27
P_Cys	0.24	0.19	0.23	0.21	T_Cys	0.23	0.19	0.22	0.21
P_Val	1.14	1.02	0.99	1.03	T_Val	1.13	1.03	0.97	1.00
P_Met	0.36	0.23	0.29	0.30	T_Met	0.34	0.22	0.29	0.31
P_Ile	1.11	1.15	0.96	1.00	T_Ile	1.10	1.14	0.95	0.97
P_Leu	2.01	1.32	1.76	1.84	T_Leu	2.01	1.38	1.71	1.78
P_Tyr	0.87	0.71	0.73	0.78	T_Tyr	0.86	0.71	0.73	0.77
P_Phe	1.15	1.36	1.00	1.04	T_Phe	1.14	1.35	0.99	1.03
P_Lys	2.18	1.43	1.84	1.97	T_Lys	2.19	1.49	1.85	1.91
P_His	0.69	0.63	0.56	0.60	T_His	0.67	0.64	0.55	0.61
P_Arg	1.65	1.50	1.40	1.52	T_Arg	1.66	1.52	1.31	1.41
P_Pro	1.01	0.98	0.84	0.97	T_Pro	1.04	0.98	0.74	0.87

Amino acid content of pectoralis and thigh were represented by P_ amino acid and T_ amino acid, respectively. Asp=Asparagine, Thr=Threonine, Ser=Serine, Glu=Glutamine, Gly=Glycine, Ala=Alanine, Cys=Cysteine, Val=Valine, Met=Methionine, Ile=Isoleucine, Leu=Leucine, Tyr=Tyrosine, Phe=Phenylalanine, Lys=Lysine, His=Histidine, Arg=Arginine, Pro=Proline. I, II, III, and IV were the four groups of the cluster analysis.

### PCA of amino acid content

**Figure-2:**
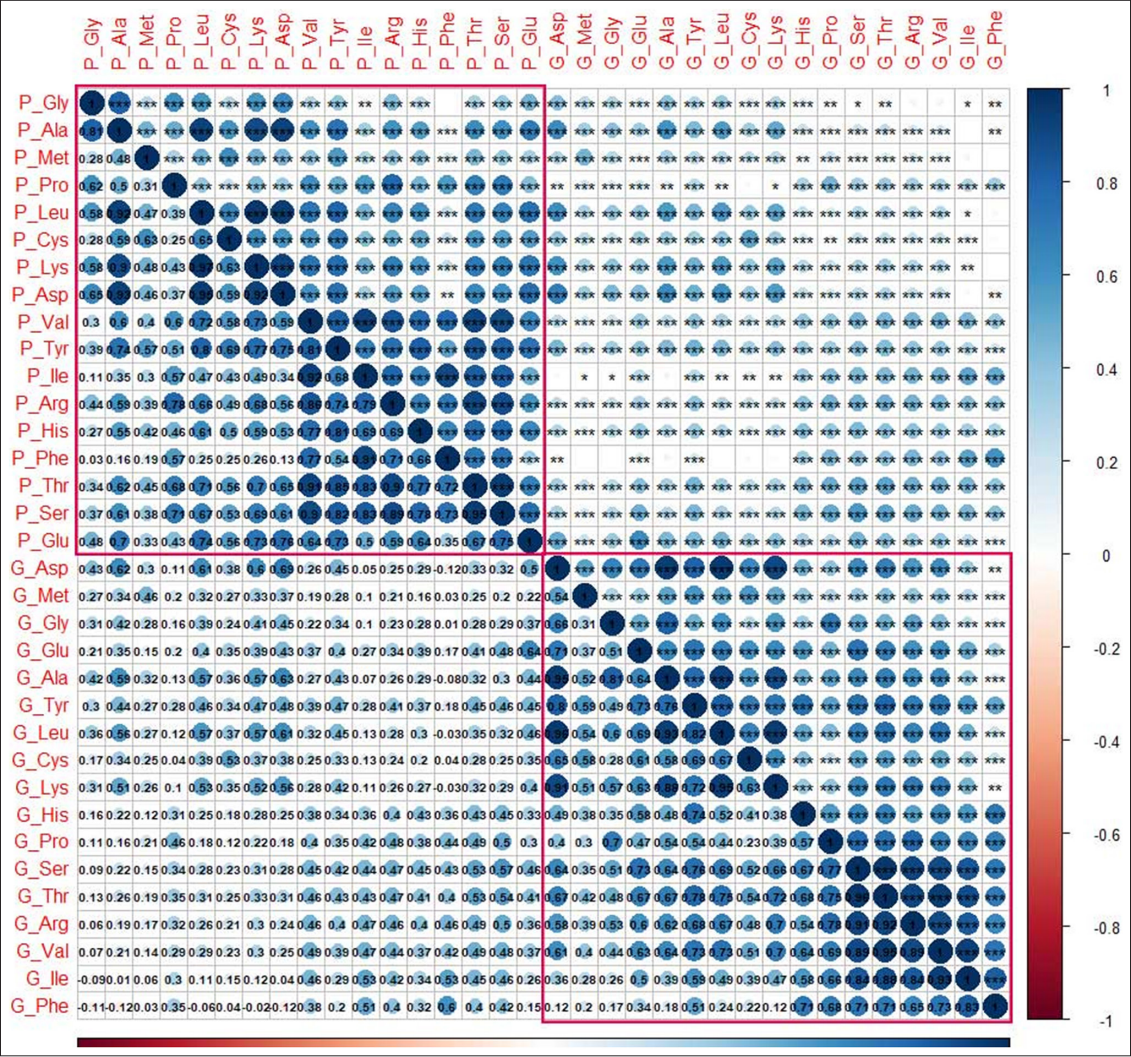
Correlation analysis of amino acid content in geese. The amino acid content of the pectoralis and thigh were represented by P_ and T_, respectively. Asp=Asparagine, Thr=Threonine, Ser=Serine, Glu=Glutamine, Gly=Glycine, Ala=Alanine, Cys=Cysteine, Val=Valine, Met=Methionine, Ile=Isoleucine, Leu=Leucine, Tyr=Tyrosine, Phe=Phenylalanine, Lys=Lysine, His=Histidine, Arg=Arginine, Pro=Proline. The number represents the correlation coefficient. *, **, and *** indicate that there was a significant correlation at p < 0.05, p < 0.01 and p < 0.001, respectively. The red rectangles on the upper left and lower right represent the correlation of amino acid content within the pectoralis and thigh, respectively.

Correlation analysis of amino acid content was performed in this study (Figure-2). Except for a negligible correlation between Gly and Phe in the pectoralis, 17 amino acids exhibited significant positive correlations with each other within the same meat cut. Furthermore, although a significant positive correlation between amino acids was observed across different meat cuts, this correlation was weaker than that observed within the same meat cut.

To comprehensively evaluate the amino acid content of the 10 germplasms, PCA was performed using data from 34 amino acids across two meat cuts. The Kaiser–Meyer–Olkin (KMO) and Bartlett tests (KMO = 0.89 > 0.65, p < 0.01) demonstrated the suitability of the data for PCA [20]. The analysis reduced the dimensionality of the 34 amino acids to five PCs, which accounted for 84.37% of the total variance, as determined by eigenvalues greater than 1 (Table-4). PC1-PC5 were subsequently selected to calculate the *D*-value for the comprehensive evaluation of amino acid content across the 10 geese germplasms. The results (Table-5) ranked the germplasms in the following order: “WX” > “TZ” > “LH” > “XP” > “GF” > “TH” > “SC” > “HY” > “YJ” > “ZD”.

**Table-4:** PCA of amino acids in 10 goose germplasms.

Items	PC1	PC2	PC3	PC4	PC5	Items	PC1	PC2	PC3	PC4	PC5
P_Asp	0.73	−0.41	0.47	−0.05	0.11	T_Glu	0.71	0.32	0.13	0.07	0.23
P_Thr	0.82	0.40	−0.29	0.03	0.01	T_Gly	0.58	0.26	0.30	-0.36	0.05
P_Ser	0.82	0.38	−0.32	−0.04	0.08	T_Ala	0.74	0.35	0.50	-0.13	0.03
P_Glu	0.74	0.32	0.11	0.00	0.28	T_Cys	0.58	0.30	0.30	0.45	-0.14
P_Gly	0.45	0.37	0.39	−0.51	-0.22	T_Val	0.77	0.51	-0.22	0.05	0.06
P_Ala	0.70	0.48	0.45	−0.16	-0.02	T_Met	0.50	0.22	0.28	0.16	-0.61
P_Cys	0.59	0.37	0.19	0.47	-0.10	T_Ile	0.63	0.50	-0.48	0.09	0.03
P_Val	0.78	0.44	−0.33	0.11	0.09	T_Leu	0.77	0.39	0.44	0.03	0.08
P_Met	0.47	0.31	0.17	0.26	-0.53	T_Tyr	0.79	0.39	0.15	0.06	-0.12
P_Ile	0.65	0.35	−0.58	0.13	0.06	T_Phe	0.49	0.42	-0.65	-0.03	-0.14
P_Leu	0.75	−0.47	0.35	0.04	0.13	T_Lys	0.71	0.38	0.44	0.06	0.10
P_Tyr	0.80	0.44	−0.02	0.15	0.04	T_His	0.64	0.34	-0.20	-0.05	-0.12
P_Phe	0.51	−0.26	−0.75	0.06	0.00	T_Arg	0.76	0.49	-0.23	-0.03	0.05
P_Lys	0.76	−0.45	0.32	−0.01	0.09	T_Pro	0.65	0.36	-0.32	-0.35	-0.11
P_His	0.71	0.37	−0.24	0.09	0.12	Eigenvalues	16.32	5.26	4.57	1.39	1.15
P_Arg	0.76	0.41	−0.33	−0.11	-0.08						
P_Pro	0.56	−0.36	−0.32	−0.45	-0.33	% variance	47.99	15.48	13.43	4.09	3.37
T_Asp	0.74	0.32	0.56	−0.03	0.08						
T_Thr	0.81	0.51	−0.18	−0.01	0.02	Cumulative %	47.99	63.47	76.90	80.99	84.37
T_Ser	0.79	0.49	−0.22	−0.03	0.10						

The amino acid content of pectoralis and thigh were represented by P_ and T_, respectively. Asp=Asparagine, Thr=Threonine, Ser=Serine, Glu=Glutamine, Gly=Glycine, Ala=Alanine, Cys=Cysteine, Val=Valine, Met=Methionine, Ile=Isoleucine, Leu=Leucine, Tyr=Tyrosine; Phe=Phenylalanine, Lys=Lysine, His=Histidine, Arg=Arginine, Pro=Proline, PCA=Principal component analysis

**Table-5:** Comprehensive evaluation and ranking of the amino acid content of 10 goose germplasms.

Germplasm	*D* ^1^	Ranking	Germplasm	*D*	Ranking
WX	0.5078	1	HY	0.4050	6
TZ	0.4970	2	TH	0.3901	7
LH	0.4639	3	SC	0.3899	8
XP	0.4258	4	YJ	0.3741	9
GF	0.4203	5	ZD	0.3570	10

1 D=Comprehensive evaluation value, GF=Guangfeng goose, HY=Huoyan goose, LH=Lianhua goose, SC=Sichuan goose, TH=Taihu goose, TZ=Taizhou goose, WX=Wanxi goose, XP=Xupu goose, YJ=Youjiang goose, ZD=Zhedong goose

## Discussion

The 10 geese germplasms encompass various types, including meat, egg, feather, and liver, with some serving dual purposes (meat: TZ, LH, GF, YJ, and ZD; meat + feather: WX and SC; meat + liver: XP; meat + egg: TH; egg + liver: HY) [8, 21, 22]. Because of their diverse regional origins, the genetic background and diversity of the germplasm in this study were abundant, as evidenced by the Shannon–Wiener Diversity Index. Therefore, this study offers the potential to screen goose germplasm resources and develop new varieties with superior meat quality. In addition, GF, YJ, and LH are cultivated in <30,000 units/year on the market, indicating concern over the current status of the breed. Hence, an accurate assessment of amino acid content in this study could aid in conservation efforts [17].

Proteins in food are absorbed as amino acids. Humans require eight EAAs that must be obtained through diet. This study detected seven EAAs, excluding tryptophan caused by acid hydrolysis. The AASs (exceeding the FAO/WHO recommended values) indicate that goose meat is a high-quality protein source with a balanced amino acid profile [16]. As the primary UTAAs, Glu and Asp were most abundant in goose meat, consistent with Zhang’s findings [15]. This suggests that goose meat has a desirable flavor. Lys, which is abundant in goose meat (only lower than Glu and Asp), is the first limiting amino acid in rice and wheat. Given Chinese dietary habits (rice and wheat as the staple food), incorporating goose meat is beneficial [16]. Met + Cys were the first limiting amino acids in goose meat, as also noted in the native Polish goose breed [23]. Fortunately, diet composition and rearing patterns can influence amino acid content, potentially increasing the concentration of limiting amino acids [15, 24, 25].

In line with a previous study by Li *et al*. [26], the amino acid content in the pectoralis was generally higher than that in the thigh, indicating superior nutritional value and flavor in the pectoralis. According to the EAA/TAA and AAS values, the amino acid composition in the thigh was more balanced. In addition, the interaction between germplasm and meat cut leads to differences in amino acid content in meat cut across germplasms, likely due to differences in amino acid metabolism pathways [27]. To date, accurate data comparing amino acid content among multiple Chinese geese germplasms are lacking. In this study, WX and TZ stood out for their superior nutritional value and flavor. Han *et al*. [28] also reported that the TAA content in WX was higher than that in ZD, consistent with the findings of this study.

Cluster analysis grouped germplasms with similar genetic information, reflecting their genetic relationships [29]. In this study, the 10 germplasms were classified into four groups. WX and TZ, exhibiting the highest amino acid content, were placed in Cluster I. In general, the cluster analysis results aligned with the geographical origins of the germplasms, suggesting a correlation between amino acid content and geographical location. However, the amino acid content of goose meat is not solely dependent on geographic origin. Specifically, TH and HY (East China) were clustered in cluster II (Southwest China) and cluster III (Central China), respectively, which might be due to the introduction of variety. Notably, TZ and TH were not clustered together despite TH being one of TZ’s progenitors, possibly due to the influence of the Rhineland goose on TZ.

Despite extensive research on differences in amino acid content among meat cuts [2, 30–32], correlations between these differences have been rarely reported. This study revealed a significant positive correlation between amino acids and meat cuts. The amino acid contents of the two meat cuts were collectively considered for a comprehensive evaluation. Because of the varying performances of the 10 germplasms on various amino acids, the amino acid contents of the two meat cuts were collectively considered for a comprehensive evaluation. Therefore, the amino acid contents of the two meat cuts were collectively considered for a comprehensive evaluation [33]. The ranking of the 10 Chinese native geese germplasms is as follows: “WX” > “TZ” > “LH” > “XP” > “GF” > “TH” > “SC” > “HY” > “YJ” > “ZD”.

## Conclusion

Overall, this study is the first to reveal diversity and systematically evaluate the amino acid content of 10 Chinese native geese germplasms. The results showed that WX and TZ germplasms had the best nutritional value and flavor. However, this study only focuses on the phenotypic differences in amino acids among these 10 geese germplasms. Future research should aim to identify the key genes and elucidate the underlying molecular mechanisms that contribute to these differences. This study provides valuable data for the conservation of existing goose germplasms and the cultivation of new breeds of goose with improved meat quality.

## Authors’ Contributions

HC and ZY: Conceptualization. HC and ZY: Methodology. HZ and ZY: Software. LW, MZ, and GS: Validation. HC and ZY: Formal analysis. XL, YB, and SR: Investigation. HZ and JW: Data curation. HC: Writing–original draft preparation. JY: writing–review and editing. XH and HG: Visualization. LW and JW: Supervision. JW: Project administration. All authors have read and approved the final manuscript.

## References

[1] Liu H.W, Zhou D.W (2013). Influence of pasture intake on meat quality, lipid oxidation, and fatty acid composition of geese. J. Anim. Sci.

[2] Yin L.Q, Xu M.X, Huang Q.K, Zhang D.H, Lin Z.Z, Wang Y, Liu Y.P (2023). Nutrition and flavor evaluation of amino acids in Guangyuan grey chicken of different ages, genders and meat cuts. Animals.

[3] Duan Y.H, Zheng C.B, Zheng J, Ma L, Ma X.R, Zhong Y.Z, Zhao X.C, Li F.N, Guo Q.P, Yin Y.L (2023). Profiles of muscular amino acids, fatty acids, and metabolites in Shaziling pigs of different ages and relation to meat quality. Sci. China Life Sci.

[4] Shabbir M.A, Raza A, Anjum F.M, Khan M.R, Suleria H.A (2015). Effect of thermal treatment on meat proteins with special reference to heterocyclic aromatic amines (Haas). Crit. Rev. Food Sci. Nutr.

[5] Millward D.J (2012). Amino acid scoring patterns for protein quality assessment. Br. J. Nutr.

[6] Spanier A.M, Flores M, Toldrá F, Aristoy M.C, Bett K.L, Bystricky P, Bland J.M (2004). Meat flavor:Contribution of proteins and peptides to the flavor of beef.*Adv*. Exp. Med. Biol.

[7] Zhou C.Y, Wang C, Tang C.B, Dai C, Bai Y, Yu X.B, Li C.B, Xu X.L, Zhou G.H, Cao J.X (2019). Label-free proteomics reveals the mechanism of bitterness and adhesiveness in Jinhua ham. Food Chem.

[8] Sun A, Wu W, Soladoye O.P, Aluko R.E, Bak K.H, Fu Y, Zhang Y (2022). Maillard reaction of food-derived peptides as a potential route to generate meat flavor compounds:A review. Food Res. Int.

[9] Ardö Y (2006). Flavour formation by amino acid catabolism. Biotechnol. Adv.

[10] Tian Y, Zhang R.K, Li G.Q, Zeng T, Chen L, Xu W.W, Gu T.T, Tao Z.R, Du X.Z, Lu L.Z (2024). Microbial fermented feed affects flavor amino acids and yolk trimethylamine of duck eggs via cecal microbiota-yolk metabolites crosstalk. Food Chem.

[11] Okruszek A, Wołoszyn J, Haraf G, Orkusz A, Wereńska M (2013). Chemical composition and amino acid profiles of geese muscles from native Polish breeds. Poult. Sci.

[12] Zhang S.Y, Zhang J.Q, Cao C, Cai Y.J, Li Y.X, Song Y.P, Bao X.Y, Zhang J.Q (2022). Effects of different rearing systems on Lueyang black-bone chickens:Meat quality, amino acid composition, and breast muscle transcriptome. Genes (Basel).

[13] Weng K.Q, Huo W.R, Gu T.T, Bao Q, Hou L.E, Zhang Y, Zhang Y, Xu Q, Chen G.H (2021). Effects of marketable ages on meat quality through fiber characteristics in the geese. Poult. Sci.

[14] Wang J.W (2015). The Current Situation, Progress and Direction of the Excavation and Utilization of Geese Genetic Resources in China. Tai'an, Shandong, China. In:6^th^ China Waterfowl Development. Conference.

[15] Zhang J, Wu Q.S, Jie X.D, Cheng Y.T, Mu Z.P, He H, Liu A.F (2020). Effects of different dietary fiber source on the meat quality and amino acid content of Sichuan white geese. China Anim. Husb. Vet. Med.

[16] Yan X.X, Xu Y.G, Zhen Z.Y, Li J.J, Zheng H.B, Li S.H, Hu Q.Q, Ye P.F (2023). Slaughter performance of the main geese breeds raised commercially in China and nutritional value of the meats of the geese breeds:A systematic review. J. Sci. Food Agric.

[17] Chen G.H, Liu J.L, Xu Q (2022). Current status and prospects of conservation and utilization of geese genetic resources in China. Guide Chin. Poult.

[18] Chen T, Liu L, Zhou Y.L, Zheng Q, Luo S.Y, Xiang T.T, Zhou L.J, Feng S.L, Yang H.Y, Ding C.B (2023). Characterization and comprehensive evaluation of phenotypic characters in wild *Camellia oleifera* germplasm for conservation and breeding. Front. Plant. Sci.

[19] Yang H, Wu Y.Q, Zhang C.H, Wu W.L, Lyu L.F, Li W.L (2022). Comprehensive resistance evaluation of 15 blueberry cultivars under high soil pH stress based on growth phenotype and physiological traits. Front. Plant. Sci.

[20] Hu M.T, Tian H.J, Yang K.Z, Ding S.Q, Hao Y, Xu R.H, Zhang F.L, Liu H, Zhang D (2024). Comprehensive evaluation and selection of 192 maize accessions from different sources. Plants (Basel).

[21] Wang H.N, Zhao H.C, Wang J, Gong D.Q (2024). Early growth pattern and growth curve fitting of Youjiang geese. Heilongjiang Anim. Sci. Vet. Med.

[22] Li X.Y, Qiu X.H, Chen C.Y, Yu X.M, Liu L.X, Lan L.T (2012). Principal component analysis of body weight and body measurement of Guangfeng white geese. China Anim. Husb. Vet. Med.

[23] Gumułka M, Połtowicz K (2020). Comparison of carcass traits and meat quality of intensively reared geese from a polish genetic resource flock to those of commercial hybrids. Poult. Sci.

[24] Wang L.W, Su S.F, Zhao J, He X.L, Fu S.Y, Wang B, Wang Y.F, Wang D.Q, Yun N.N, Chen X, Belobrajdic D.P, Terigele Li X.D, Jiang L.L, He J.F, Liu Y.B (2023). Effects of dietary oat supplementation on carcass traits, muscle metabolites, amino acid profiles, and its association with meat quality of Small-tail Han sheep. Food Chem.

[25] Wang B, Wang Y.J, Zuo S.X, Peng S.J, Wang Z.J, Zhang Y.J, Luo H.L (2021). Untargeted and targeted metabolomics profiling of muscle reveals enhanced meat quality in artificial pasture grazing tan lambs via rescheduling the rumen bacterial community. J. Agric. Food Chem.

[26] Li Z.L, Chen L, Du X, Wu H.Z, Shen J.L, Shi F.X, Lu L.Z (2019). Comparison of meat quality of F1 geese generated with different cross combinations. Chin. J. Anim. Sci.

[27] Chen G.H, Qi L, Zhang S, Peng H.Q, Lin Z.T, Zhang X.Q, Nie Q.H, Luo W (2024). Metabolomic, lipidomic, and proteomic profiles provide insights on meat quality differences between Shitou and Wuzong geese. Food Chem.

[28] Han F, Wang F, Li P, Zhou H, Li C, Xu B.C (2023). Comparison on chemical composition and quality characteristic of meat from different geese breeds. Food Res. Dev.

[29] Dalmaijer E.S, Nord C.L, Astle D.E (2022). Statistical power for cluster analysis. BMC Bioinf.

[30] Oh M, Kim E.K, Jeon B.T, Tang Y, Kim M.S, Seong H.J, Moon S.H (2016). Chemical compositions, free amino acid contents and antioxidant activities of Hanwoo (*Bos taurus* coreanae) beef by cut. Meat Sci.

[31] Ou H.M, Zhang X.L, Tan Z.L, He L.Q, Jiao J.Z, Li J.Z (2022). Evaluation of muscle quality and analysis of nutrient composition in different parts of Hulun Buir lambs. Chin. J. Anim. Nutr.

[32] Gui G.B, Zeng Z, Hu G.F, Xiao X.Y, Wu W.J, Chen P, Xia Y.J, Zhang H.Q (2023). Comparison of meat quality in different parts of castrated cattle. Feed Res.

[33] Jolliffe I.T, Cadima J (2016). Principal component analysis:A review and recent developments. Philos Trans. A Math. Phys. Eng. Sci.

